# Barriers to Antiretroviral Therapy Adherence in Rural and Urban Areas in Indonesia: Perspectives of People Living with HIV and Healthcare Professionals

**DOI:** 10.3390/tropicalmed11080220

**Published:** 2026-08-07

**Authors:** Nelsensius Klau Fauk

**Affiliations:** AXIS Research Health Institute, Torrens University Australia, Adelaide 5000, Australia; nelsensius.fauk@torrens.edu.au

**Keywords:** barriers, antiretroviral therapy, people living with HIV, rural and urban areas, Indonesia

## Abstract

Antiretroviral therapy (ART) is essential for preventing HIV transmission and improving the health outcomes of people living with HIV (PLHIV). However, many barriers limit PLHIV from starting and adhering to ART, which explains why HIV responses in many settings, including Indonesia, have produced limited gains. This qualitative phenomenological study explored multilevel barriers to ART adherence in urban Yogyakarta (locally known as Jogja) and rural Belu, Indonesia, from the perspectives of PLHIV and healthcare professionals (HCPs). Data were collected through one-on-one in-depth interviews with 92 PLHIV and 20 HCPs. Participants were recruited using the snowball sampling technique. Data were analysed using framework analysis informed by the Access to Healthcare Framework. The findings showed that PLHIV in Belu and Jogja had different experiences in terms of the provision of and ability to access and adhere to ART or HIV treatment. In rural Belu, ART was less available and visible, harder to approach, often unaffordable, less aligned with patients’ needs, and strongly influenced by the widespread use of traditional medicine. PLHIV in Belu also reported a more limited ability to perceive the need for ART, reach services, pay costs, engage in care, and seek ART than those in urban Jogja. Personal, psychological, and social barriers were also reported to hinder PLHIV’s ART adherence in both settings. These findings highlight the need for HIV policies that promote the equitable distribution of ART services and targeted interventions to improve understanding and acceptance of HIV care among PLHIV and the wider community.

## 1. Introduction

The scale-up of HIV treatment, commonly known as antiretroviral therapy (ART), has been highly effective in sustaining viral suppression, preventing HIV transmission, and improving health outcomes for people living with HIV (PLHIV), thereby reducing HIV-related morbidity and mortality [1,2]. However, global progress remains below the UNAIDS 95-95-95 target [3]. Of an estimated 40.9 million PLHIV in 2025, only 87% knew their HIV status; among those who knew their status, only 89% accessed ART, and among those receiving ART, 94% achieved viral suppression [1]. These figures indicate persistent gaps in ART coverage and suggest uneven treatment outcomes across subpopulations and geographical areas, particularly in rural settings [2]. In Indonesia, HIV remains a major public health challenge. HIV infections and AIDS-related deaths have increased substantially over the past decade, underscoring the importance of expanding ART coverage and sustaining effective ART among people who have initiated treatment [4]. Of an estimated 564,000 PLHIV in Indonesia, only 63% knew their HIV status, 67% of those diagnosed accessed ART, and 55% of those on ART achieved viral load suppression [4,5]. For PLHIV receiving ART, consistent adherence is essential to achieve and maintain viral suppression and the individual and public health benefits of treatment. Although first-line HIV treatment is provided free of charge by the government, excluding administrative and related service costs such as clinic registration fees [6], Indonesia continues to face challenges in sustaining engagement in care and long-term ART adherence among PLHIV who have initiated treatment [4].

ART engagement and adherence can be understood as the result of interacting barriers and facilitators operating across individual, interpersonal, community, health-system, and structural levels. At the individual level, systematic reviews have identified barriers such as forgetting doses, being away from home, alcohol or substance use, treatment-related side effects, limited treatment literacy, competing work and caregiving responsibilities, and mental health challenges [7,8]. These factors can disrupt the knowledge, motivation, stability, and daily routines required for consistent medicine-taking, timely refills, and continued adherence [7,8]. At the interpersonal and community levels, HIV-related stigma, fear of disclosure, secrecy, reduced social support, and avoidance of local services are repeatedly associated with poorer ART adherence and retention in care [7,8,9]. Stigma can generate shame, isolation, and fear of being identified as HIV-positive, discouraging PLHIV from seeking support or using nearby services [7,8,9]. Health-system and structural barriers also affect sustained ART engagement and adherence, including unavailable or distant services, shortages of HIV-trained healthcare professionals (HCPs), medication stock-outs, and financial hardship related to transport, clinic fees, or other care-related costs [7,8,9]. These barriers can delay medicine collection, interrupt care, or lead to treatment discontinuation despite ongoing clinical need [7,8,9].

Studies in Indonesia similarly show that barriers to ART occur at multiple levels. At the intrapersonal level, limited knowledge of HIV, ART services, and treatment effectiveness has been reported as a barrier to ART engagement among Indonesian PLHIV [10,11,12,13,14,15]. Other intrapersonal barriers include misconceptions that treatment is unnecessary in the absence of symptoms, fear of side effects or dependence on medication, depression and anxiety, the use of traditional medicine, alcohol or injecting drug use, imprisonment, and self-stigma [11,12,14,16]. Social barriers include concerns about confidentiality breaches; religious beliefs; discouragement from relatives, neighbours, or friends; and issues related to transgender identity [11,13,14,15,17]. Community and healthcare-related stigma, moral judgement, and discriminatory norms may also disrupt adherence and continuity of care by encouraging concealment and social isolation [18,19,20]. At the health-system and structural levels, Indonesian studies have identified limited or unavailable ART services, complex clinic procedures, negative provider attitudes, poor access to health insurance, financial difficulties, inadequate service linkages, rural residence, and long travel distances to HIV clinics as key barriers to ART engagement [13,14,15,16,20,21].

Despite this evidence, important gaps remain. First, much of the existing research has focused on single geographical contexts, limiting the understanding of how multilevel barriers differ between rural and urban areas within the same national setting [10,20,21,22,23]. Second, although some studies have examined either PLHIV (demand side) or HCP (supply side) perspectives, fewer have explicitly explored these perspectives together to triangulate how barriers are experienced by service users and produced or reinforced within health systems and communities [11,20]. Third, previous Indonesian studies have documented many barriers, but less is known about how these barriers interact across the supply-side and demand-side dimensions of healthcare access. Importantly, because the present study recruited PLHIV through HIV clinics and clinic-linked networks, all PLHIV participants had already initiated ART. The study therefore focuses on barriers to ongoing ART adherence and continuity of care, rather than barriers to HIV testing, diagnosis, linkage, or ART initiation. PLHIV who were not attending clinics or had never initiated ART may experience additional or different barriers and are not represented in this study. The present study, therefore, compares how barriers to ART are configured differently in rural and urban contexts and interprets these differences through a health-access framework. This triangulated approach is intended to provide more context-sensitive evidence for HIV treatment policy and practice. Therefore, this study aims to explore multilevel barriers to ART adherence and continuity of care based on the perspectives of PLHIV and HCPs in rural and urban areas of Indonesia. By integrating these perspectives, the study contributes to a more triangulated understanding of ART adherence barriers and provides evidence to inform more responsive HIV treatment interventions.

### Conceptual Framework

This study used the Access to Healthcare Framework [24] to explore factors that influence ART adherence among PLHIV in urban Jogja and rural Belu. The framework conceptualises access as an interaction between the supply side (e.g., HCPs, organisations, and systems) and the demand side, represented in this study by PLHIV [24,25,26]. When these dimensions function effectively, they can facilitate sustained ART adherence; when they are absent or insufficient, they may become barriers.

The framework proposes five supply-side dimensions of access: availability, approachability, affordability, appropriateness, and acceptability. These correspond to five demand-side abilities: the ability to reach, perceive, pay, engage, and seek care [24,25,26]. Availability refers to the presence of ART services, resources, and trained healthcare providers and is linked to PLHIV’s ability to reach services, which may be influenced by transport, mobility, work schedules, and time. Approachability concerns whether information about ART services is accessible and understood, corresponding to PLHIV’s ability to perceive their need for ART and know where and how to obtain it. Affordability refers to the direct and indirect costs of care, including medical, transport, and other related expenses, and corresponds to PLHIV’s ability to pay. Appropriateness reflects the extent to which ART services meet PLHIV’s needs and provide an acceptable quality of care, supporting their ability to remain engaged in and adhere to treatment. Acceptability refers to the social, cultural, and interpersonal factors shaping whether ART services are considered acceptable and is linked to PLHIV’s ability to seek care, including their understanding of services and rights to access them.

## 2. Methods

The reporting of the study’s methods and findings was guided by the Consolidated Criteria for Reporting Qualitative Research (COREQ) checklist [27]. COREQ comprises 32 items (Figure S1) designed to promote transparent, thorough, and comprehensive reporting of qualitative studies.

### 2.1. Study Design and Participant Recruitment

Data presented in this paper were drawn from a large-scale qualitative phenomenological research project exploring HIV-related risk factors, impacts, and barriers to ART engagement from the perspectives and experiences of PLHIV and HCPs in urban Jogja and rural Belu, Indonesia. This design was appropriate because the study aimed to gain in-depth insight into participants’ perspectives, experiences, and interpretations of factors influencing their ART journeys [28,29]. It also enabled the exploration of similarities and differences between an urban setting and a rural setting, while still preserving the depth of individual accounts.

Jogja and Belu were purposively selected as contrasting service contexts. Jogja represents a more urbanised setting in which HIV-related services were available through several hospitals, *Puskesmas*, and HIV clinics with established peer-support and outreach networks [30]. Belu represents a rural district in eastern Indonesia in which villages are geographically dispersed, and HIV services were concentrated in a hospital-based HIV clinic [31]. This contrast enabled the study to examine how barriers to ART engagement operated differently in an urban and a rural area rather than assuming that barriers were uniform across Indonesia.

Participants included men and women living with HIV and HCPs providing HIV care services in the two study settings. PLHIV were eligible if they were aged 18 years or older, had been diagnosed with HIV, and were able to participate in an interview in Indonesian. HCPs were eligible if they were directly involved in HIV testing, treatment, counselling, ART dispensing, referral, or related HIV service delivery in one of the study settings. Including both groups enabled the study to capture complementary demand-side and supply-side perspectives and supported triangulation, thereby strengthening the depth, credibility, and comprehensiveness of the findings. Participants were recruited using snowball sampling, which was considered appropriate given the sensitivity of HIV-related research, the vulnerability of PLHIV, and the persistent stigma surrounding HIV in the study settings [18,32,33]. However, it is acknowledged that this approach may have limited participant diversity and led to the recruitment of PLHIV who had engaged in ART, as discussed in the Section 4.2. The recruitment started with the distribution of study information sheets through HIV clinic receptionists and clinic information boards in both districts. This strategy was used to identify initial participants, after which snowball sampling was applied to recruit other participants through participant referrals. The information sheets included the researcher’s contact details. Potential participants who contacted the researcher and expressed interest were invited for an interview at a mutually agreed time and location. Two potential participants withdrew shortly after their interviews began for personal reasons, and their data were excluded from the analysis. PLHIV were eligible if they were aged 18 years or older, had been diagnosed with HIV, and were able to participate in an interview voluntarily. HCPs were eligible if they were directly involved in HIV testing, treatment, counselling, ART dispensing, referral, or related HIV service delivery in one of the study settings. Recruitment took six months. In total, 92 PLHIV participated, comprising 52 women and 40 men, with 46 recruited from Jogja and 46 from Belu. In addition, 20 HCPs participated and were included in the analysis.

### 2.2. Data Collection

Data were collected through one-on-one in-depth interviews. Interviews were conducted in private settings agreed upon by the researcher and participants: a rented house near the HIV clinic in Jogja and a private room at the HIV clinic in Belu. All interviews were conducted by NKF, a male researcher with a PhD in Public Health, formal training in qualitative methods, and more than 10 years of experience conducting public health research, especially research on HIV/AIDS with various marginalised and underserved population groups. Before each interview, participants were informed that ethics approval had been obtained from committees in Australia and Indonesia. They received a verbal explanation of the study’s purpose and the use of the data or information provided in the interview. They were also informed about the voluntary nature of their participation and were assured about the confidentiality and anonymity of the data or information they provided in the interview. Written or verbal consent, including consent for audio recording, was obtained from all participants. Only the researcher and each participant were present during the interviews. Interviews were conducted in Indonesian, the national language spoken in both study sites and the researcher’s first language. Field notes were taken when necessary. The interview guide was informed by the Access to Healthcare Framework and explored participants’ views and experiences of barriers to ART engagement. Questions focused on the five supply-side dimensions of access and the five corresponding demand-side abilities. Interviews lasted between 35 and 87 min and were digitally audio-recorded. No repeat interviews were conducted. Recruitment, data collection, transcription, and preliminary coding were conducted concurrently, enabling saturation to be monitored iteratively throughout fieldwork. Emerging codes and themes were compared across participant groups (PLHIV and HCPs), study settings (rural Belu and urban Jogja), and the supply-side and demand-side dimensions of the Access to Healthcare Framework. Recruitment and data collection continued until the researcher judged that the data were sufficiently rich to address the research aim and that data saturation had been reached. Saturation was indicated by recurring information across the final interviews, with no substantial new insights emerging [34]. No repeat interviews were conducted with any of the participants. The opportunity to read and correct the interview transcripts was not offered to participants living with HIV due to the sensitivity of the topic of HIV and to prevent the possibility of transcripts being received and read by their family members, who could reveal the participants’ HIV status if they had not already disclosed it to family members. This opportunity was offered to HCP participants, but none of them took advantage of it. The researcher had no prior relationship with any participant.

### 2.3. Data Analysis

Comprehensive data analysis began after the manual and verbatim transcription of the digital audio recordings and the integration of the field notes into coding sheets (NKF). After this, the transcripts of individual interviews were imported into NVivo 12 for comprehensive data analysis. Framework analysis for qualitative data by Ritchie and Spencer [35] was used to guide the entire data analysis process, which followed the five steps offered in the framework. The first step involved familiarisation with individual transcripts (data) through repeatedly reading each transcript, breaking down the data into small chunks, and making comments or labels on the data extracts of each transcript from rural Belu and urban Jogja. Prior to this formal analysis, the researcher had engaged with individual transcripts repeatedly during the transcription and integration of field notes into each transcript. Initial identification of similarities and differences in barriers to ART between the two settings was also performed. The second step involved the identification of a thematic framework, which was deductively guided by the concepts from the Access to Healthcare Framework and prior knowledge of the topic and inductively emerged from the data. This step began with the identification of key issues and concepts that recurrently emerged from the data that were collected based on the comments and labels made in the first step. Themes used to form the thematic framework were also derived from the Access to Healthcare Framework concepts and purely emerged from the data. The identification of the thematic framework was an iterative process that involved changing and refining themes. The third step involved indexing the data employing both deductive and inductive approaches. Data indexing or coding started with performing open coding, where codes or notes were provided to data extracts in each interview transcript. This was followed by close coding to identify and group similar or redundant codes or nodes, which were then used to form themes. Deductive themes informed by the Access to Healthcare Framework included availability, reachability, and the awareness of ART services; affordability and the ability to pay for ART services; and service appropriateness, acceptability, and the ability to engage in ART. Inductive themes that fell outside the framework were personal, psychological, and social barriers to ART engagement and traditional medicines and approvals from others. For example, codes regarding the provision of ART services in both rural and urban settings, sources and dissemination of ART information, and the ability of PLHIV in both settings to reach or access the services in healthcare facilities and HIV clinics and perceive the need for ART were grouped and presented under the theme “Availability, reachability and the awareness of ART services”. Codes regarding feelings of boredom, fatigue, and being burdened with ART; emotional distress; fear of death and social rejection; HIV stigma and discrimination were grouped and presented under the theme “Personal, psychological, and social barriers to ART engagement”. The fourth step was charting the data. This was done by reorganising and summarising codes from each interview transcript, which had been grouped into separate themes in the previous section, and grouping them under each theme. This enabled comparison across interviews and within each interview. The fifth step involved mapping and interpreting the data, where key characteristics of the data were pulled together, mapped, and interpreted as a whole [35,36]. The framework helped the management of qualitative data in a coherent and structured way and guided the analytic process in a rigorous, transparent and valid way. Coding and analysis were conducted in Indonesian, and quotes used in this manuscript for publication purposes were translated into English. Accuracy of the translation and credibility of the findings were maintained through repeated checking and rechecking of transcripts against the translated interpretations or examination of meaning in both source (Bahasa) and target (English) languages during the analysis [37].

To strengthen transparency in a single-author analysis, the analytic process moved repeatedly between framework-guided categories and inductively generated codes. PLHIV and HCP accounts were compared within and across rural Belu and urban Jogja, and contrasting accounts were retained in the analytic matrices so that differences between settings and participant groups could be traced. Although inter-coder reliability was not possible, credibility was supported through repeated transcript reading, maintenance of coding sheets, comparison of translated quotations against Indonesian transcripts, and explicit use of participant roles and settings when presenting quotations.

To contextualise the interview excerpts and avoid reducing participants to demographic labels, each quoted participant is presented using a pseudonym created for reporting, followed by a concise descriptor such as role; gender, where relevant; marital status when already analytically meaningful; and rural or urban setting. These pseudonyms are not real names and are not linked to clinic records. Where more than one excerpt came from the same participant, the same pseudonym is used throughout the manuscript.

## 3. Results

### 3.1. Sociodemographic Profile of the Participants

A total of 92 women and men living with HIV and 20 HCPs were interviewed in both study settings. The majority of participants were in the age group of 30–49 years and were married (see Table 1). The 92 participants had been living with HIV for varying durations, with 55 individuals reporting living with the infection for 1–5 years, 25 individuals reporting 6–10 years, and 12 individuals reporting 11–15 years. Among the 20 HCPs, 10 had been involved in HIV healthcare service delivery for 1–5 years, while the remainder had been engaged for either 6–10 years or 11–15 years. In terms of education, 55 out of the 92 women and men living with HIV reported having graduated from junior or senior high school and 21 from university, while the rest had either completed primary school or had dropped out of school. Of the 20 HCPs, 17 held a bachelor’s degree, 2 had master’s degrees, and 1 held a PhD. The 92 women and men living with HIV reported participating in various types of employment, with 21 women identifying as housewives (see Table 1).

### 3.2. Availability, Reachability and the Awareness of ART Services

As all participants in both settings had already been receiving ART, their accounts reflected how the availability and approachability of ART services shaped their ability to reach ART services and perceive the need for the treatment. In Jogja, both PLHIV and HCPs reported that ART services were available through hospitals, *Puskesmas*, and HIV clinics. Access was supported by short travel distances, flexible hours, and the availability of private and public transport, including motorbikes and buses. ART-related information was also disseminated through multiple channels, including healthcare providers, peer companions, community outreach, mobile VCT, and WhatsApp groups. Because these support systems were close to participants’ everyday routines, PLHIV in Jogja often described ART access as manageable rather than exceptional. Adi, a married man living with HIV who lived near a clinic in urban Jogja, linked service availability to short travel distances and transport options. Maya, a medical doctor involved in HIV services in Jogja, described a dense information network involving HCPs, peer companions, outreach activities, mobile VCT, and WhatsApp groups:

*“The medicines and medical tests like CD4 (cluster of differentiation 4) and viral load tests are available in hospitals and community health centres here (Jogja). …I am living nearby so I can easily reach the clinic using my motorbike or public transport”*.

*(Adi, married man living with HIV, Jogja)*

“*I think HIV-related health services in the city of Jogja are known to them (PLHIV) and general community members because information about the services is disseminated to them regularly. Healthcare providers and companions of PLHIV regularly inform people about the services, HIV information sessions, mobile VCT or through WhatsApp groups*”.

(*Maya, medical doctor, Jogja*)

In contrast, although participants in rural Belu had also been receiving ART, they described services as limited and geographically difficult to access. ART was available only at one HIV clinic attached to a public hospital in town, and only two HIV-trained doctors provided HIV care in the district. CD4 and viral load testing were unavailable locally, which concerned both PLHIV and HCPs because these tests were important for monitoring treatment progress. Several PLHIV reported delaying or missing medicine collection as ART was unavailable at nearby *Puskesmas*. This problem was compounded by long travel distances, difficult terrain, a lack of public transport, and high transport costs. Markus, a married man living with HIV in rural Belu who did not own a motorbike, described how dependence on an expensive ojek could turn medicine collection into an irregular and burdensome journey:

*“Public transport from my house to the community health centre and to this clinic is not available. I do not have a motorbike. This is difficult for me, and I feel burdened every time I come here to collect medicine because I have to use a motorbike taxi (Ojek), and it is expensive. So, sometimes I come (for ART), sometimes I don’t”*.

(Markus, married man living with HIV, Belu)

HCPs in Belu confirmed these barriers from the supply side. Frans, a medical doctor, described the local HIV service package as restricted mainly to HIV testing and ART provision, while CD4 and viral load tests were not available. Nurse Yohana, who provided HIV counselling in Belu, emphasised how long distances and a lack of transport reduced patients’ ability to collect medication regularly and remain engaged in care. These accounts suggest that ART engagement in Belu was constrained not only by individual motivation but also by health-system and structural barriers, particularly limited service capacity, geographic inaccessibility, and unaffordable transport:

*“HIV care services available here (in Belu) are HIV testing and antiretroviral medicines, only these two items. The medicines are available in this (HIV) clinic, so all patients (PLHIV) access the medicines here. It is not possible to do CD4 and viral load tests here (HIV clinic)…”*.

(Frans, medical doctor, Belu)

“*Many patients (PLHIV) here live far away from this community health centre and the HIV clinic in town, … so the distance to this community health centre and HIV clinic is quite far. Public transportation is not available here, and even in some areas here, motorbike taxi is not available either*”.

(Yohana, nurse and counsellor, Belu)

Differences were also evident in the dissemination of ART information (approachability aspect of the ART services). In Jogja, ART services were actively promoted through health workers, peer supporters, outreach activities, mobile VCT, and digital communication. In Belu, however, information about ART often reached PLHIV only after they or their spouses became ill, were admitted to the hospital, and were diagnosed with HIV. HCPs were often the only source of ART information, and limited community participation in HIV information sessions further restricted awareness. Ester, an HCP in Belu, explained that many patients in Belu learnt about HIV treatment only after being hospitalised and testing positive, at which point counsellors then informed them about ART:

*“Only a small number of people attend activities (HIV information sessions and mobile VCT) that we carry out. That is why HIV services and information are still unknown to many people. Patients (PLHIV) here started to know about HIV and HIV treatment or ART once they were sick, admitted to this hospital and tested positive with HIV, and then the counsellor informed them about the treatment or ART”*.

(Ester, nurse and counsellor, Belu)

Despite these barriers, PLHIV in both settings generally understood the importance of ART and expressed a need to continue treatment. This perception was reinforced by support from HCPs and peer companions, who provided reminders and encouragement. In Jogja, Sari, a married woman living with HIV, included ART collection and monitoring as part of her normal routine. In Belu, Lukas, a married man living with HIV, described continued engagement as closely linked to provider-led reminders and encouragement. These findings show that sustained ART engagement was shaped by the interaction between service availability, geographic access, information dissemination, personal recognition of treatment need, and ongoing support from HCPs and companions:

*“I remember the date I have to collect the medicines every month and the month I have to check my viral load and CD4. These are just like my daily need for food”*.

(Sari, married woman living with HIV, Jogja)

“*I come here to collect the medicines every month because I need them. The doctors and nurses always remind and encourage me to collect the medicines on time here …*”.

(Lukas, married man living with HIV, Belu)

### 3.3. Affordability and the Ability to Pay for ART Services

Affordability shaped PLHIV’s ability to remain engaged in ART across both study settings. Most participants in Jogja and some in Belu reported that ART was financially accessible because they were covered by National Health Insurance, either through the fully government-subsidised Indonesian Health Card (Kartu Indonesia Sehat/KIS) or the independent BPJS scheme, which required monthly contributions. For these participants, health insurance reduced or removed treatment-related costs, while stable income helped cover insurance fees and transport to HIV clinics. As Maria, a married woman living with HIV in rural Belu, explained, “*I use the KIS, so I do not need to pay anything, including the administration costs*”. Similarly, Budi, a married man living with HIV in urban Jogja, noted that insurance and transport costs were manageable because both he and his wife had income:

*“I pay … one hundred and twenty thousand rupiahs for me and my wife (the monthly fee of the insurance, second class). I can still handle it because I work (have income) and my wife also has income, … For transport, we spend about fifty thousand rupiahs on gasoline for my motorbike…”*.

(Budi, married man living with HIV, Jogja)

However, affordability remained a major barrier for nearly half of the women and more than half of the men interviewed in Belu, as well as a few participants in Jogja. These participants described difficulty paying insurance contributions, clinic-related expenses, and transport costs. Financial hardship was closely linked to unemployment, unstable income, and dependence on family or community support. When such support was uncertain or unavailable, participants felt insecure and were at risk of delaying or interrupting ART. One participant in Jogja explained that although he was currently supported by people at a mosque, he worried about being unable to continue paying BPJS fees because he had no income. In Belu, Rosa, a married woman living with HIV, described repeated delays in medicine collection when transport money was unavailable:

*“Sometimes, if I do not have money at all, then I am not able to come to collect the medicines here (VCT clinic). I was late collecting the medicines for a few days several times because I did not have money for the transportation costs”*.

(Rosa, married woman living with HIV, Belu)

Economic difficulties were often intensified by the consequences of HIV itself. Some participants reported that illness reduced their ability to work or forced them to stop working, while household healthcare expenses increased. The financial impact was particularly severe when spouses were also living with HIV, placing additional pressure on families. In some cases, participants or their relatives sold assets, depleted savings, or borrowed money to cover daily needs and healthcare costs. Ana, a married woman in Belu whose husband had stopped working as an ojek driver, explained that the household had no income while still needing to provide food, drinks, and support for their children:

*“At the moment we (the woman and her husband) do not have income at all because my husband has stopped ‘Ojek’ (motorbike taxi driver). I have not worked so far; … We need food and drinks, and we need to provide for the children. These are very much burdensome for our family”*.

(Ana, married woman living with HIV, Belu)

HCPs in both settings confirmed that financial insecurity undermined ART engagement. In Belu, limited financial resources prevented some PLHIV from paying for transport, registration fees, medical tests, and BPJS contributions, which delayed treatment initiation or contributed to treatment interruption. In Jogja, Dewi, a medical doctor, noted that students from outside Java were sometimes late collecting medicines when family financial support was delayed. These findings show that although health insurance facilitated ART access for many participants, financial insecurity, transport costs, and unstable income remained important structural barriers to sustained treatment engagement:

“*There are patients who do not collect antiretroviral medicines because they do not have money to pay for transport costs. They are mostly single and university students from outside of Java. Their life depends on the support from their parents, so if their parents have not sent them money or were late sending them a monthly living allowance, then they will be late to collect the medicines here (community health centre)*”.

(Dewi, medical doctor, Jogja)

### 3.4. Service Appropriateness, Acceptability, and Ability to Engage in ART

The appropriateness and acceptability of ART services influenced participants’ ability to engage in and seek ongoing treatment. In Jogja, both PLHIV and HCPs reported that HIV care services generally met patients’ needs. In addition to ART, supporting tests such as viral load, CD4 count, and kidney and liver function tests were available, enabling patients and providers to monitor treatment effectiveness. Participants also described service delivery as appropriate because care was provided by trained HCPs in a supportive and professional manner. Lestari, a remarried woman living with HIV in Jogja, explained that the availability of ART together with viral load, CD4, kidney, and liver function tests met her main treatment needs: “*What I need is the medicines and the tests like viral load, CD4, and kidney and liver function tests. All these services are provided*”. Wulan similarly noted that HIV services in Jogja were relatively complete, including ART and key laboratory monitoring:

*“I would say HIV-related health services for patients (PLHIV) here (Jogja) are complete, and patients continue to access them until now. ….”*.

(Wulan, medical doctor, Jogja)

In contrast, participants in rural Belu described HIV care as incomplete. Although ART was available, CD4 and viral load testing were not provided locally, limiting patients’ ability to monitor their treatment progress. Both PLHIV and HCPs viewed this as a major gap in service appropriateness. Antoni, a married man living with HIV in Belu, stated, “*The healthcare services are not complete in this clinic. We need those tests, but we cannot do the tests here*”. Frans confirmed that ART was the only HIV treatment service available and that the absence of CD4 and viral load testing meant the service did not fully meet patients’ needs:

*“The service does not meet the needs of the patients because other medical tests, like CD4 and viral load tests, are not available. …”*.

(Frans, medical doctor, Belu)

Despite differences in service completeness, participants in both settings generally perceived ART services as acceptable. They described HCPs providing HIV healthcare services as kind, encouraging, and supportive and reported trusting relationships with those providing care. These positive interactions supported their willingness to seek and remain engaged in ART. Participants also demonstrated knowledge of where ART services were available, indicating an ability to seek care. However, ART engagement was still constrained by limited family support and the need to conceal HIV status. Some participants reported stopping or interrupting treatment because family members did not accept their HIV status, were unaware of their diagnosis, or were physically distant. Paulus, a man living with HIV in Belu, explained, “*I did not receive any support from my family, which is why I sometimes stopped treatment*”. Melati, a single woman living with HIV in Jogja, similarly described carrying the burden alone after hiding her HIV status from her family, which contributed to non-adherence. These accounts suggest that while respectful and supportive care from HCPs enhanced acceptability, social isolation and a lack of family support continued to undermine sustained engagement in ART:

“*I hid my status from my parents and siblings, who all live in the village. So I have had to carry this burden alone. I often did not adhere to treatment because I felt so alone*”.

(Melati, single woman living with HIV, Jogja)

### 3.5. Personal, Psychological, and Social Barriers to ART Engagement

HCPs across both study settings reported that treatment non-adherence among PLHIV was influenced not only by external barriers but also by personal and psychological factors. In Belu, boredom and fatigue from taking lifelong medication appeared to reduce motivation to continue ART, indicating the burden of long-term treatment on everyday life. In Jogja, emotional distress following diagnosis, particularly the fear of death and social rejection, seemed to discourage some patients from seeking care and starting treatment. Frans and Lina described these barriers from their clinical and counselling experience. Their accounts indicate that PLHIV’s ART engagement can be undermined by both treatment fatigue and the psychological impact of living with HIV, highlighting the importance of ongoing emotional and adherence support:

*“There are patients who feel lazy and bored with antiretroviral medicines and do not want to adhere to the treatment. Some said ‘I feel very bored to take the medicines every day’”*.

(Frans, medical doctor, Belu)

“*There are patients who struggle at the beginning of the diagnosis and are psychologically depressed due to the fear of death and fear of being ostracised or avoided by others. Such psychological pressures and fears make them isolate themselves from others and unwilling to access health services or undergo HIV treatment*”.

(Lina, nurse and counsellor, Jogja)

HIV stigma and discrimination were also reported to impede individuals’ engagement with ART services, which were corroborated by HCPs interviewed in both Jogja and Belu. Their accounts suggest that stigma and discrimination remained powerful barriers to ART access and adherence by creating fear, shame, and mistrust around HIV care. Concern about negative treatment from both HCPs and community members appeared to discourage some PLHIV from disclosing their status, seeking care, or initiating treatment. Yosef, a single man living with HIV in Belu, described both community and healthcare-related rejection, while Nurse Rini, an HCP in Jogja, explained how anticipated stigma discouraged service use. These accounts indicate that stigma was not only anticipated but also directly experienced in healthcare and social settings, likely reinforcing the avoidance of services. This highlights how HIV-related stigma can operate across interpersonal, community, and institutional levels to undermine timely and sustained engagement in treatment:

*“I had stopped treatment (ART) for a while because I experienced people around me, my family and neighbours, not wanting to be close to me. Even at the hospital, I feel the same thing”*.

(Yosef, single man living with HIV, Belu)

“*Stigma and discrimination against them (PLHIV) still occur and affect their access to HIV care services. They fear stigma and discrimination from healthcare providers who do not understand HIV or from community members if their HIV status is discovered. …*”.

(Rini, nurse and counsellor, Jogja)

### 3.6. Traditional Medicines and Approvals from Others

The narratives from participants in rural Belu indicated that the acceptability of ART services was influenced by the practice of traditional HIV treatments using traditional medicines provided by local healers, a factor not reported by participants in Jogja. Antoni, a married man living with HIV in Belu, and Elena, a remarried woman living with HIV in Belu, both described stopping ART after diagnosis and using traditional medicines instead. Their accounts show that reliance on traditional practices could hinder the early initiation of ART and affect adherence to therapy:

“*After I was diagnosed with HIV, I started the treatment (ART), but I didn’t continue it and used traditional medicines instead. I came back here (to restart ART) a few months ago because I felt like my health condition was not getting better*”.

(Antoni, married man living with HIV, Belu)

*“I stopped taking medicines from here (ART from the clinic) and continued the treatment using traditional medicines from traditional healers”*.

(Elena, remarried woman living with HIV, Belu)

Furthermore, participants, both women and men living with HIV, described that their use of traditional medicines was supported by their family members, relatives, neighbours, and friends. This suggests that the use of traditional medicines was shaped not only by individual preference but also by strong social influence from family members and the wider community. Daniel, a single man living with HIV in Belu, described how neighbours encouraged his parents to support traditional treatment even after he had started ART. Advice and encouragement from parents and neighbours appeared to legitimise traditional treatment as a better option, even after ART had been initiated, which could weaken confidence in biomedical care and interrupt treatment continuity. The account highlights how close social networks can act as a barrier to ART when they promote alternative forms of care that compete with or discourage adherence to HIV treatment:

*“Our neighbours told my parents, and they encouraged us that traditional medicines are actually better, and this disease should be treated with traditional medicines. I had started taking antiretroviral medicines, but my parents encouraged me to use traditional medicines. ….”*.

(Daniel, single man living with HIV, Belu)

The utilisation of traditional medicines by individuals living with HIV in rural Belu was also highlighted by HCPs interviewed in this context. Nurse Kornelis, a counsellor in Belu, described traditional medicine use as closely linked to limited ART knowledge. Their accounts suggest that the use of traditional medicines alongside or in place of ART was influenced by both a limited understanding of HIV treatment and strong family involvement in decision-making. Patients who lacked adequate knowledge about ART and depended heavily on family support appeared more vulnerable to following relatives’ preferences, including switching to traditional remedies. This indicates that treatment adherence was shaped not only by individual beliefs but also by family dynamics and patients’ dependence on others, making family influence a significant barrier to sustained ART use:

*“Many (HIV) patients use traditional medicines even though they have started ART, they can easily switch to traditional treatment. Their family members play an important role in it. The patients do not have much knowledge about ART, and mostly they are dependent on their family support, so I can see that it is highly likely that they go along with what their family members suggest”*.

(Kornelis, nurse and counsellor, Belu)

## 4. Discussion

This study explores the views and experiences of PLHIV and HCPs in rural Belu and urban Jogja regarding PLHIV’s engagement in HIV treatment or ART. The findings reveal urban–rural disparities in engagement with ART and suggest that ART engagement did not depend only on the willingness of PLHIV to start or continue treatment; it emerged from the fit between the supply side of care (availability, approachability, affordability, appropriateness, and acceptability) and the demand-side abilities of PLHIV to reach, perceive, pay, engage, and seek care [24]. Nevertheless, these findings should be interpreted within the specific social, geographic, and healthcare contexts of rural Belu and urban Jogja, as well as the characteristics of the study participants, particularly PLHIV who were already linked to HIV treatment networks in both settings. In urban Jogja, the interaction between multiple service points, HIV-trained HCPs, outreach initiatives, peer support, and monitoring tests created a relatively enabling treatment environment. In rural Belu, a single ART site in the town, limited HIV-trained staff, long travel distances to the clinic, limited transport options, limited monitoring capacity, financial insecurity, stigma, and traditional medicine use interacted to constrain ART engagement. Thus, the findings extend the Indonesian literature by showing not only which barriers exist but also how they combine differently across rural and urban contexts and across service-user and provider perspectives [20,21,22]. The contrasts observed between these two locations reinforce global evidence that ART engagement is influenced not only by individual motivation but also by health system capacity that is geographically patterned, as well as factors such as poverty and stigma—elements that contribute to unequal progress towards global HIV treatment and viral suppression targets [38,39,40].

This study shows that the availability and approachability of ART services and the corresponding abilities to reach care and perceive the need for ongoing treatment strongly shaped PLHIV’s ART engagement [24]. In urban Jogja, ART services were available through hospitals, *Puskesmas*, and HIV clinics and were supported by shorter travel distances, transport options, proactive information dissemination, peer support, and digital communication. These conditions increased service visibility, improved ART literacy, and supported linkage and continuity of care. Similar urban Indonesian studies among men who have sex with men and transgender women have shown that visible services, outreach, peer support, and trusted providers can facilitate HIV care engagement, although stigma and confidentiality concerns remain persistent barriers [14,22,41,42]. The findings are also consistent with evidence that decentralised services, community outreach, and civil society partnerships strengthen access to or engagement of PLHIV in HIV treatment [20,43,44,45,46,47,48]. In contrast, ART services in rural Belu were centralised in a single hospital-based HIV clinic and were not supported by CD4 and viral load testing. Long distances, poor transport, travel costs, and limited community-based HIV information made ART less reachable and less visible for many PLHIV, echoing findings among Papuans living with HIV and broader evidence that centralised services, weak transport systems, limited health communication, and low HIV literacy undermine testing, linkage, retention, and adherence, particularly in rural and remote settings [7,46,49,50]. The policy implication is that service availability must extend beyond medicine supply to include decentralised refills, referral pathways, and monitoring, counselling, and accessible information. Decentralising ART initiation and refills to *Puskesmas*, strengthening task-sharing and mentoring, and introducing community-based pick-up points, outreach distribution, peer navigation, and multi-month dispensing for stable patients could reduce the travel burden while normalising early and lifelong ART engagement [39,45,48,51,52,53].

The affordability of ART services and the ability to pay are also crucial aspects that determine the engagement and adherence of PLHIV to the therapy [24]. Although first-line ART was described as free or subsidised, the findings show that treatment can remain economically inaccessible when indirect costs are high. In Jogja, most participants could manage costs because clinics were closer and income or insurance helped cover expenses, although students and participants concealing their HIV status from family remained financially vulnerable. In Belu, however, affordability was tightly connected to geography: transport to the town clinic, registration fees, BPJS contributions, and loss of income created recurrent risks of late collection or interruption. This finding aligns with global evidence that “free ART” does not eliminate financial barriers when transport, missed work, food insecurity, and ancillary medical costs remain substantial [7,46]. It also supports evidence that HIV and poverty can reinforce one another: illness reduces work capacity while treatment-related and household costs increase, potentially producing debt, asset sales, and dependence on others [54]. Policy responses should therefore treat ART affordability as more than the medicine price. Transport support, flexible clinic hours, locally available medicine refills, social protection linkages, and support for insurance enrolment or premium payment are likely to be especially important in rural and low-income contexts [55].

This study also highlights the appropriateness and acceptability of ART services, as well as individuals’ ability to engage in and seek these services, as factors influencing PLHIV’s engagement in ART [24]. In Jogja, the appropriateness of the service was reflected in the availability of ART alongside viral load, CD4, kidney, and liver function tests, which, together with high-quality service delivery by trained HCPs, helped meet PLHIV’s clinical needs and strengthened confidence in treatment. In Belu, ART provision without local CD4 and viral load testing represented a system-level gap because patients could not easily monitor treatment progress or treatment failure, potentially weakening confidence in ART when symptoms persisted or recurred. This aligns with WHO guidance emphasising comprehensive HIV service delivery and routine monitoring across the HIV care continuum [39], while also showing that integration or service presence alone may not always improve adherence unless the service package meets patients’ practical and clinical needs [56,57]. The finding that HCPs were generally described as kind, supportive, and trustworthy is important because patient-centred care can counterbalance wider stigma and sustain willingness to remain in care [41,58]. However, supportive provider relationships were not sufficient when patients lacked family support, concealed their HIV status, or feared social rejection. This suggests that ART services should include adherence counselling that addresses disclosure concerns, family dynamics, confidentiality, and psychosocial support rather than focusing only on medicine collection.

The current findings also suggest that across both settings, engagement was further affected by individual and social barriers, including treatment fatigue, work commitments, side effects, limited ART literacy, psychological distress after diagnosis, and HIV-related stigma. These findings are consistent with studies in Indonesia and other contexts showing that fear, self-stigma, anticipated discrimination, and poor treatment literacy can delay ART initiation and disrupt adherence [7,12,20,59,60]. These findings highlight the importance of certain stigma-reduction interventions (e.g., cognitive–behavioural components, empowerment, etc.) that can improve both psychosocial wellbeing and ART adherence, as previously reported elsewhere [61,62]. The Belu findings add an important rural and cultural dimension: traditional medicines were often used alongside or instead of ART, and this decision was often endorsed by family members, neighbours, and friends. This reflects medical pluralism, in which biomedical and traditional care coexist and compete, and is consistent with evidence from other populations that traditional and complementary treatment can interrupt ART when it is preferred by patients or their social networks [63,64]. The implication is not that traditional beliefs should be dismissed, but that HIV programmes should engage them respectfully. Community dialogue, family-inclusive counselling, collaboration with trusted local actors, and clear education about ART effectiveness and the risks of delayed treatment may help reduce treatment interruption while avoiding further stigma toward people who use or value traditional care. Stigma-reduction interventions in healthcare and community settings, particularly those combining empowerment, cognitive–behavioural approaches, contact with PLHIV, and provider training, should also be considered because such interventions can improve psychosocial wellbeing and treatment engagement [61,62].

### 4.1. Researcher Reflexivity

Acknowledging the researcher’s role in knowledge production is essential in qualitative research, as it helps identify potential biases, recognise the influence of personal experience and interpretation, and enhance the credibility of the findings. The researcher’s background in public health, HIV research, health services research, and qualitative methods shaped the design, conduct, and interpretation of this study. His prior experience with HIV-related stigma and healthcare access informed the development of interview questions, particularly the focus on multilevel barriers, service access, stigma, and social support. During interviews, the researcher’s familiarity with HIV issues helped create a sensitive and respectful interview environment and enabled probing of difficult topics such as disclosure, stigma, treatment interruption, and traditional medicine use. However, it may also have influenced how participants presented their experiences, particularly if they perceived the researcher as knowledgeable about HIV services. To minimise this, participants were reminded that there were no right or wrong answers and were encouraged to describe their own experiences in their own words. Reflexivity was also applied during analysis. The researcher repeatedly checked emerging interpretations against interview transcripts and field notes, compared narratives across PLHIV and HCPs and across rural and urban settings, and remained attentive to accounts that challenged or complicated the dominant patterns. The selection of excerpts was guided by their relevance to the themes, their ability to illustrate variation across settings and participant groups, and their connection to the framework, rather than by their agreement with the researcher’s expectations. This process helped ensure that analytical priorities reflected participants’ narratives and the study’s aim, while acknowledging that interpretation was inevitably shaped by the researcher’s disciplinary background and position.

### 4.2. Limitations and Strengths of the Study

The findings should be interpreted with caution due to several limitations. First, the use of snowball sampling and recruitment through HIV clinics may have introduced selection bias. These strategies likely drew participants (PLHIV) from similar social networks and disproportionately included those already engaged in care and ART. As a result, PLHIV not on ART, who may have different views and experiences regarding barriers to ART, were likely under-represented. Similarly, among HCP participants, only those directly involved in HIV service delivery were recruited. Providers who are not directly engaged in HIV care and who may hold different perspectives or have different experiences regarding barriers to ART were therefore undersampled. This may have limited the breadth of perspectives captured and may have resulted in an incomplete understanding of barriers to ART from both PLHIV and HCP viewpoints. Therefore, the findings primarily reflect the experiences and perspectives of the participants included in this study, which may differ from those of other PLHIV and HCPs in settings with different characteristics. Another limitation is that because coding and analysis were conducted by one researcher, inter-coder reliability and researcher triangulation were not possible. As a result, the development of codes and themes reflected a single analytic perspective, and no cross-checking of disagreements in interpretation could be performed. In addition, consistent with the nature of qualitative research, this study was not designed to claim statistical generalisability in the way quantitative studies with representative samples can. Nonetheless, the study has important strengths. It contributes to a better understanding of how multilevel barriers differ between rural and urban areas within the same national context, an area where evidence remains limited, particularly in Indonesia. Furthermore, by explicitly presenting and triangulating the perspectives of PLHIV (demand side) and healthcare providers (supply side), the study triangulates how constraints and enabling conditions are experienced and shaped across communities and the health system. The depth and richness of these findings can help inform ART-related policy and practice in the study sites and in other similar settings in Indonesia and beyond.

## 5. Conclusions

This study presents the views and experiences of PLHIV who had been receiving ART and HCPs regarding barriers to ART in urban Jogja and rural Belu, Indonesia. The findings show some differences in ART supply-side dimensions between the two settings. In rural Belu, ART services were far more constrained across the dimensions of availability, approachability, affordability, appropriateness, and acceptability. Unlike urban Jogja, ART services in rural Belu were limited, less visible and accessible, often unaffordable, less aligned with patients’ needs, and strongly shaped by the widespread use of traditional medicine. Differences were also observed in PLHIV’s abilities to perceive, reach, pay for, engage with, and seek ART services. Participants in rural Belu generally reported more limited capacity in these areas than those in urban Jogja. These limitations were influenced by multiple factors, including long travel distances, a lack of public transportation, unemployment and low income, dependence on family and others for support, and pressure from family members, relatives, neighbours, and friends to use traditional medicines provided by local healers. Personal, psychological, and social barriers also hindered PLHIV’s ART engagement in both settings. The findings support rural-sensitive HIV policies that decentralise ART and monitoring services, reduce indirect treatment costs, strengthen peer and community support, address stigma, and engage family and community influences on treatment decisions. Future large-scale quantitative studies are recommended to examine various factors affecting both ART supply and PLHIV’s ability to use ART services. Evidence from such research could guide tailored interventions and improve ART service delivery for PLHIV, particularly in rural areas of Indonesia and similar contexts globally.

## Figures and Tables

**Table 1:** Sociodemographic profile of the participants.

Characteristics	Women with HIV		Men with HIV		Healthcare Professionals	
	Jogja (n = 26)	Belu (n = 26)	Jogja (n = 20)	Belu (n = 20)	Jogja (n = 10)	Belu (n = 10)
**Age**						
18–29	6	6		7		
30–39	12	12	10	5	2	4
40–49	8	6	10	5	6	4
>50		2		3	2	2
**Marital status**						
Single/never married	5	3	5	7	2	3
Divorced	5	1	2			
Widowed/r	3	12		1		
(Re)Married	13	10	13	12	8	7
**HIV diagnosis**						
1–5 years ago	16	18	6	15		
6–10 years ago	7	7	7	4		
11–15 years ago	3	1	7	1		
**Involvement in HIV services**						
1–5 years					4	6
6–10 years						4
11–15 years					6	
**Religion**						
Islam	23	1	17		5	
Catholic/Protestant	3	25	3	20	5	10
**Education**						
Doctorate/postgraduate					3	
University graduate/diploma	6	6	7	2	7	10
Senior high school graduate	13	5	11	8		
Junior high school graduate	6	6	2	4		
Elementary school graduate/dropout	1	9		6		
**Occupation**						
Housewife	10	11				
Entrepreneur	3	6	10	1		
Tailor, laundress, shopkeeper	3					
Sex worker	1					
Teacher, police, farmer				6		
Nurse/health worker/NGO worker	4	2				
Private employee, civil servant/retired	3	4	5	2		
University student	1			1		
Taxi/truck/motorbike driver, mechanic			2	10		
Unemployed	1	3	3			

## Data Availability

The data presented in this study are available on request from the corresponding author. The data are not publicly available due to restrictions set by the human research ethics committee.

## Supplementary Materials

The following supporting information can be downloaded at: https://www.mdpi.com/article/10.3390/tropicalmed11080220/s1, Figure S1: COREQ (COnsolidated criteria for REporting Qualitative research) Checklist.

## Abbreviations

The following abbreviations are used in this manuscript:

ART	Antiretroviral Therapy
BPJS	*Badan Penyelenggara Jaminan Sosial* (Social Security Agency for Health)
CD4	Cluster of Differentiation 4
HIV	Human Immunodeficiency Virus
KIS	*Kartu Indonesia Sehat* (Indonesian Health Card)
NGOs	Non-Governmental Organisations
PLHIV	People Living with HIV
HCPs	Healthcare Professionals
